# Mathematical modelling for pandemic preparedness in Canada: Learning from COVID-19

**DOI:** 10.14745/ccdr.v50i10a03

**Published:** 2024-10-03

**Authors:** Nicholas H Ogden, Emily S Acheson, Kevin Brown, David Champredon, Caroline Colijn, Alan Diener, Jonathan Dushoff, David JD Earn, Vanessa Gabriele-Rivet, Marcellin Gangbè, Steve Guillouzic, Deirdre Hennessy, Valerie Hongoh, Amy Hurford, Lisa Kanary, Michael Li, Victoria Ng, Sarah P Otto, Irena Papst, Erin E Rees, Ashleigh Tuite, Matthew R MacLeod, Carmen Lia Murall, Lisa Waddell, Rania Wasfi, Michael Wolfson

**Affiliations:** 1 Public Health Risk Sciences Division, National Microbiology Laboratory, Public Health Agency of Canada; 2 Public Health Ontario, Toronto, ON; 3 Dalla Lana School of Public Health, University of Toronto, Toronto, ON; 4 Department of Mathematics, Simon Fraser University, Burnaby, BC; 5 Health Policy Branch, Health Canada, Ottawa, ON; 6 Department of Biology and Michael G. DeGroote Institute for Infectious Disease Research, McMaster University, Hamilton, ON; 7 Department of Mathematics and Statistics and Michael G. DeGroote Institute for Infectious Disease Research, McMaster University, Hamilton, ON; 8 Centre for Operational Research and Analysis, Defence Research and Development Canada, Department of National Defence, Ottawa, ON; 9 Health Analysis Division, Analytical Studies and Modelling Branch, Statistics Canada, Ottawa, ON; 10 Department of Biology and Department of Mathematics and Statistics, Memorial University of Newfoundland, St. John’s, NL; 11 Department of Zoology and Biodiversity Research Centre, University of British Columbia, Vancouver, BC; 12 Centre for Immunization Programs, Public Health Agency of Canada, Ottawa, ON; 13 Public Health Genomics Division, National Microbiology Laboratory, Public Health Agency of Canada; 14 Faculty of Medicine and Faculty of Law-Common Law, University of Ottawa, Ottawa, ON

**Keywords:** mathematical modelling, pandemic preparedness, infectious diseases, COVID-19

## Abstract

**Background:**

The COVID-19 pandemic underlined the need for pandemic planning but also brought into focus the use of mathematical modelling to support public health decisions. The types of models needed (compartment, agent-based, importation) are described. Best practices regarding biological realism (including the need for multidisciplinary expert advisors to modellers), model complexity, consideration of uncertainty and communications to decision-makers and the public are outlined.

**Methods:**

A narrative review was developed from the experiences of COVID-19 by members of the Public Health Agency of Canada External Modelling Network for Infectious Diseases (PHAC EMN-ID), a national community of practice on mathematical modelling of infectious diseases for public health.

**Results:**

Modelling can best support pandemic preparedness in two ways: 1) by modelling to support decisions on resource needs for likely future pandemics by estimating numbers of infections, hospitalized cases and cases needing intensive care, associated with epidemics of “hypothetical-yet-plausible” pandemic pathogens in Canada; and 2) by having ready-to-go modelling methods that can be readily adapted to the features of an emerging pandemic pathogen and used for long-range forecasting of the epidemic in Canada, as well as to explore scenarios to support public health decisions on the use of interventions.

**Conclusion:**

There is a need for modelling expertise within public health organizations in Canada, linked to modellers in academia in a community of practice, within which relationships built outside of times of crisis can be applied to enhance modelling during public health emergencies. Key challenges to modelling for pandemic preparedness include the availability of linked public health, hospital and genomic data in Canada.

## Introduction

The COVID-19 pandemic, caused by SARS-CoV-2, underlined the need for planning for future pandemics. There have been multiple pandemic preparedness initiatives at national and international levels ((1,2)). Modelling has supported previous pandemic plans, and the World Health Organization (WHO) has included modelling as a source of evidence to support planning ((3)). In Canada, modelling supported decisions during the pH1N1 pandemic ((4–6)) and subsequent pandemic influenza planning ((7)). During the COVID-19 pandemic, the role of modelling to support decisions was brought into focus. Mathematical models synthesize information on disease transmission in the population, disease severity in different age or population groups, population immunity, effectiveness of non-pharmaceutical interventions (NPIs) and vaccine effectiveness, among other aspects. In so doing, models produce a narrative that is interpretable by decision-makers and the public, and supports evidence-based decision-making, transparency and public trust.

The objective of this article is to describe how modelling efforts can support pandemic preparedness, including a description of the model types, their roles, best practices for their use and the expertise that is required, as informed by past pandemics and our recent experiences with COVID-19. For this article, modelling is considered to include mathematical and simulation approaches to understanding and predicting the introduction, invasion, spread, evolution and control of pandemic-causing pathogens, as well as impacts on healthcare capacity. The focus is on preparedness for pathogens that spread in the human population via human-to-human transmission, with the capability of dispersing through the global travel network. It is likely that such pathogens would emerge from animal reservoirs by zoonotic transmission. While spill back to animal reservoirs, as has occurred with SARS-CoV-2, may be a feature of the transmission and ecology of such pathogens, the significance for pandemic preparedness depends on its impact on human-to-human transmission (i.e., whether ongoing animal-human contact causes the basic reproduction number *R*_0_ to be greater than one). WHO has produced a list of priority pathogens based on their importance to public health, but their criteria are broad and go beyond the capacity to cause a pandemic ((8)), so this list is too long to consider in its entirety for pandemic planning. For example, zoonoses including MERS-CoV, Nipah, Crimean-Congo haemorrhagic fever and Rift Valley fever are listed, though they often have limited human-to-human transmission, complex transmission cycles and routes of spillover into human populations involving arthropod vectors and wild and domesticated animal reservoirs. Outbreaks of these diseases may be defined by WHO as pandemics because they affect multiple countries. However, the absence of sustained human-to-human transmission, or conditions for zoonotic transmission in Canada, means that, without further evolution, they are unlikely to cause outbreaks in Canada at the scale of COVID-19 or the 1918 and 2009 influenza pandemic, for which pandemic planning aims to prepare us. Modelling supports our understanding of the potential risk from these diseases, particularly in the context of climate change ((9)), but that is out of scope for this article.

## Methods

A narrative review of how modelling can best support pandemic preparedness was developed by members of the Public Health Agency of Canada External Modelling Network for Infectious Diseases (PHAC EMN-ID), a national community of practice on mathematical modelling of infectious diseases for public health. Authors sought and reviewed scientific papers and grey literature published in the last 20 years on pandemic preparedness in Canada, and the use of modelling during the COVID-19 pandemic. As most authors were involved in modelling to support decisions at federal, provincial and/or territorial levels, their expert opinion and lived experiences on how modelling supported public health decision-makers during the pandemic were captured.

## Results

There are two components to modelling support for pandemic preparedness:

1. Modelling the transmission of ”hypothetical-yet-plausible” pandemic pathogens to support decisions on preparatory activities, such as emergency stockpiles

2. Developing validated modelling methods and tools that are maintained, capable of rapid adaptation to the biology of emerging pathogens and are thus ”ready-to-go” to support decisions in the event of the emergence of a pathogen with pandemic potential

In either case, there are considerations of good practices for modelling methods, communication of the results of modelling and data needs for modelling. People trained to recognize and fill modelling needs, embedded with, or having strong relationships with, public health organizations and decision-makers are also essential ((10)). While not explicitly a part of pandemic planning, modelling can also support resilience to pandemics, which is discussed at the end of this article.

### Good practices

Mathematical models currently used to support public health and health policy decisions need to balance biological realism with tractability ((11,12)). While models should be simple enough to understand and implement efficiently ((13)), useful realism involves incorporating the biological processes of infection and recovery, outcomes of infection, human behaviour that underpins pathogen transmission and effectiveness of NPIs and pharmaceutical interventions (see below). However, the more complex a model, the more prone it is to undetected errors and inaccurate parameterization ((13–15)). Overcomplexity may also limit standard model-evaluation methods, such as sensitivity analyses ((16)) and the ability for models to be calibrated to data ((17)). However, developments in computing power, data availability and synthesis increasingly allow tractable modelling based on transmission with a digital twin of society to model social contacts in detail ((18)). Outputs of very simple models can also have value as an adjunct to communicating aspects of an emerging epidemic to a lay audience, which may be the public or non-expert managers, as was the case during the COVID-19 pandemic ((19)). Development of criteria that can be universally used to distinguish “good models” from “bad models,” discussed as verification and validation of modelling in the broader simulation literature ((20,21)), remains a work in progress ((11,12,22)).

### Types of models

The main model types relevant to pandemic preparedness are 1) dynamic transmission models of spread of an infectious pathogen within a population, both for prediction, assessment of alternative response strategies and impact of evolutionary changes; 2) importation models that explore the estimated risk of disease importation into and within Canada based on the global network of air and land travellers and knowledge of transmission in source countries ((23)); and 3) geographic spread models that are capable of identifying spatial pathways of pathogen spread within Canada ((24)).

Dynamic transmission models typically divide human (or animal) populations into ”compartments,” such as susceptible-exposed-infectious-recovered (SEIR) models. Flows (or transitions) among these compartments reflect the fundamental processes of the biology of transmission, infection and recovery. They are described by event rates, which can be used to define deterministic, continuous flows between compartments or stochastic transitions.

The simplest SEIR models assume that the population mixes homogeneously. As a result, these models usually overestimate the spread of infections, including the peak size of epidemics. Age-based contact matrices can improve these models by using the results of population surveys ((25)) or demographic data ((26)) to estimate the frequency of daily contacts between individuals. Furthermore, SEIR models can be constructed with more complexity to model different sections of the population ((27,28)) or to model variants and evolutionary changes ((29,30)).

Agent-based models (ABMs, also called individual-based models) can incorporate even more heterogeneity. Simpler ABMs are conceptually similar to SEIR models but explicitly model individuals in a population (i.e., “agents”) who exist in susceptible, exposed, infected or recovered states. Agent-based models allow the integration of contact matrices, the construction of quasi-realistic environments (e.g., home, workplace, schools, leisure venues, public transit) within and between which the agents move according to their demographics, and potentially drawing on more extensive use of socioeconomic data. This structure allows for more realistic exploration of targeted NPIs, such as limited closures ((31)), and combinations of NPIs with vaccination ((32)). Both compartmental and agent-based models can be used to study the geographic spread of an infectious disease, in which case transmission can be modelled relatively simply in each grid cell of a landscape with plausible cell-to-cell spread of infection that depends on geographic or other physical constraints ((24,33)), or more elaborately based on more detailed data synthesis including small area estimation.

At the beginning of a pandemic that has emerged in another country, importation models can be used to estimate the probability of importation and the number of cases that may have recently been introduced into Canada by points of entry ((23)). Importation models typically consider travel volumes from different countries and/or provinces, and infection prevalence and immunity within those countries and/or provinces. Importation models can also inform travel measures within a country. Once within-country transmission has begun, importation models can provide imported case input to models of community transmission ((34,35)). As seen during COVID-19, for smaller provinces and territories, importation, rather than community transmission, can be the focus. There may be relatively few travel routes into small jurisdictions, which can be monitored and managed to prevent community outbreaks, at the outset of, and during a pandemic.

Coupling the analysis of geographic spread with genomic analyses is increasingly being used to model transmission and detect sources of new cases for a variety of pathogens, most notably COVID-19 ((36–38)). A real-time practical use of these methods is Nextstrain ((39)), an open-source platform and dashboard that allows decision-makers, scientists and the general public to watch, in real time, how the virus is evolving and spreading globally. Underlying phylogeographical and phylodynamic methods are mathematical and statistical models that rely on population genetics models, Bayesian modelling and linked SEIR-type models.

There is an array of modelling and estimation tools to provide intelligence during outbreaks. These include estimation of the instantaneous reproduction number, *R_t_* ((40)), forecasting based on wastewater signals ((41,42)), branching process models to explore control methods early in outbreaks ((43–45)) and analysis of phylogenetic trees of whole genome sequence data to obtain estimates of the basic reproduction number, *R*_0_, of the pathogen and/or emerging variants to compare with estimates from surveillance data ((46)).

### Biological realism

**Figure 1:**
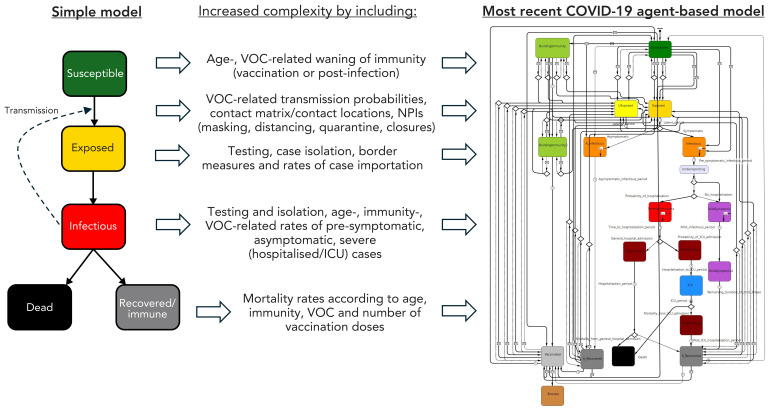
Adding complexity to a simple Susceptible-Exposed-Infectious-Recovered (SEIR) model to realistically model public health interventions^a^ Abbreviations: ICU, intensive care unit; NPIs, non-pharmaceutical interventions; VOC, variant of concern ^a^ The left hand diagram shows the structure of a simple Susceptible-Exposed-Infectious-Recovered (SEIR) model, next to which are examples of the factors that had to be introduced to realistically model COVID-19 transmission with emerging VOCs, and the use of NPIs and vaccinations, resulting in a complex model structure (right-hand diagram). In this case, the complex model is the Public Health Agency of Canada agent-based model, as described, in an earlier form, in Ng *et al*., 2020 ((30))

During the COVID-19 pandemic, the importance of models for public health decision-making strengthened, not least because it was recognized that their outputs were biologically realistic ((47)). To achieve this, the structure of models (i.e., compartments/states and flows/transitions between compartments and states) needs to be realistic in terms of 1) the biology of infection, age and sex-related likelihood of clinical outcomes and recovery (infections being asymptomatic, mild, requiring hospital, or intensive care [i.e., in ICU]), accounting for heterogeneity in different population groups where these are important in transmission, and data that are available; 2) age and sex-related patterns of contacts between infected and uninfected people, vectors or animal reservoir hosts ((48)) and how these are likely to change; 3) public health interventions (NPIs and vaccinations); and 4) where possible, realistic direct impacts on healthcare resources and indirect impacts, such as cancelled surgery and avoidance of emergency department visits (**Figure 1**). Parameter values (e.g., the duration of latent and infectious periods, the basic reproduction number, *R*_0_, contact patterns within the population) need to be realistic and obtained from prospective studies or inferred from digital twin-style data synthesis and the scientific literature using established knowledge synthesis methods ((49)). They can also be obtained by fitting models to surveillance or hospital data, particularly for parameters that are difficult to measure in studies, such as the probability of transmission when infected people contact susceptible people. The capacity to fit models to surveillance data (e.g., human cases, hospitalizations and wastewater data) depends on the availability of reliable data, which has been a problematic and largely unresolved issue during and after the COVID-19 pandemic in Canada (50). To achieve biological realism (and ideally socioeconomic realism) useful for public health objectives, modelling must be a multidisciplinary endeavour, synthesizing knowledge and data from a spectrum of scientists and clinicians involved in public health. With these principles in place, evidence provided by models will be more reliable.

### Uncertainty

In general, modelling approaches should account for plausible ranges and distributions of parameter values (e.g., the duration of infectivity) or probabilities of event occurrence (e.g., transmission probability estimates) to allow for exploration and quantification of uncertainty. There are at least three types of uncertainty to consider:

1. Data uncertainty due to measurement error

2. Uncertainty due to inherently variable parameters

3. Uncertainty as to whether the model structure fully represents the true system

Comparing initial model results to observed data may suggest that the model outcomes have very high uncertainty, and the models are insufficiently robust to support decisions without significant changes to model structure, parameters and/or calibration, in order to progress from a development stage. Validation may indicate that model results are robust enough to be useful for decision-making in two ways. If uncertainty is very small, models may have a high enough precision to say, “if public health effort is changed by X%, incidence will change by Y%.” However, models may result in outcomes that have a high degree of uncertainty, yet still have enough precision to be useful in answering less granular questions, such as, “will this be big or small?” or “is it better to do X, Y or nothing?”

### Types of modelling projects

There are two main types of predictive modelling projects: 1) forecasting and 2) scenario exploration. Forecasting is the use of mathematical models to predict the trajectory of an epidemic or outbreak in the near or far future (e.g., Slide 7 in ((51))). Scenario-based modelling is the use of models to answer “what if?” questions. Common “what if?” questions include the potential epidemiological impact (e.g., on incidence, hospitalizations, deaths) of various interventions (e.g., treatment or vaccination roll out, NPIs) ((31,52)). Scenario-based modelling often assesses outcomes over the course of multiple generations of infections. Sometimes, forecasts may also have simplified scenarios. For example, PHAC COVID-19 forecasts included a forecast of the current disease trajectory and scenarios of what might happen in a short time scale if NPIs were tightened or relaxed.

### Communicating modelling results to decision-makers and the public

Effective communication of modelling results to public health managers and decision-makers is essential. Beyond simply the general need for good oral and visual communication methods that use accessible, accurate and jargon-free language, there are some modelling-specific requirements. First, objectives of modelling need to be clear and placed in the context of decision-maker needs and, ideally, modellers, managers and decision-makers discuss and agree upon what is needed and possible at the outset ((12)). Assumptions and limitations of models, results of validation, as well as sources and degrees of uncertainty need to be communicated to clarify the degree to which model outputs are actionable by decision-makers ((11,53)). Communicating the results of models in an early stage of development and models that perform poorly in validation, as well as poorly communicated results, will likely be unproductive or even counterproductive, resulting in managers, decision-makers and stakeholders losing confidence in modelling. A further layer of care needs to be added when communicating model outcomes to the public. For example, scenario-based modelling conducted early in the COVID-19 pandemic was misinterpreted by some members of the public, press and politicians as being a forecast. When the worst-case scenario did not happen (because public health measures were implemented), there was a perception that modelling was simply wrong and that COVID-19 was overblown ((47)).

### Modelling ”hypothetical-yet-plausible” pandemic pathogens before pandemics occur

#### Objective

Pre-pandemic modelling aims to provide a foundation for decisions on pandemic planning, including the healthcare resources that need to be maintained in national stockpiles. Scenario-based modelling is needed to explore the full potential impacts on Canadian health systems of ”hypothetical-yet-plausible” pandemic pathogens in Canada. Outcomes of interest are the numbers of cases, hospitalizations, ICU treatments and deaths and the rate at which they occur. With these values, healthcare needs (hospital capacity, ICU capacity, ventilators, personal protective equipment, antivirals) can be estimated ((54)) and the quantities of healthcare stockpiles (such as the National Emergency Strategic Stockpile ((55))) and NPI measures needed, accounting for their negative health impacts, can be evaluated.

#### Models

Both compartmental models and ABMs can be used for this purpose. Agent-based models may be particularly useful to explore impacts in smaller and/or more heterogeneous communities.

#### Likely pathogens/disease types

A prioritization of likely emerging pandemic pathogens remains to be done, but zoonotic pathogens that become human-to-human transmissible by contact or the respiratory route (e.g., influenzas, coronaviruses, haemorrhagic fevers) ((56,57)) are considered likely candidates. At the time of writing, WHO is undertaking a process that is more specifically aiming for a list of priority pathogens of pandemic potential ((58)).

#### Data needs

”Most likely” epidemiological parameter values can be sourced from the literature using established knowledge synthesis methods ((49)).

### Development of modelling methods, tools and personnel ”ready-to-go” in the face of a pandemic

#### Objectives

Modelling development in the face of an epidemic should ensure that modelling methods and the necessary highly qualified personnel (HQP) are present and ready to respond to an emerging epidemic in Canada.

#### Models

Generic, adaptable and preferably validated compartmental models and ABMs need to be developed in advance so that they can be adapted to an emerging pandemic in Canada for the purpose of forecasting and conducting scenario-based modelling to guide public health interventions. Models based on a design for modelling respiratory diseases would likely be readily adaptable to other forms of direct human-to-human transmission, but it would also be valuable to have models that are more explicitly designed for a variety of transmission routes (e.g., sexual transmission ((59))). Importation models need to be ready to estimate rates and routes of importation of infectious people into Canada. Ideally, models of geographic spread within Canada, allowing exploration of interventions that limit spatial spread, would also be ready for adaptation to an emerging pathogen. There is also a need for modelling and estimation methods that enhance analysis of surveillance data, including estimation of the instantaneous reproduction number, *R_t_*, forecasting from clinical surveillance and wastewater data and assessing genomic data to provide estimates of key epidemiological parameters, including *R*_0_ and selection advantage of emerging variants.

#### Data needs

In the face of an emerging pandemic that begins outside Canada, as experienced during the COVID-19 pandemic, key parameter values for modelling emerge in the evolving scientific literature and knowledge synthesis skills are needed to be ready to source them (49). Ideally, estimates of key parameter values for a range of pathogens from the current literature would allow models to be populated with ”best estimates” at the start of a pandemic prior to quantification of parameters specific to the emerging pandemic. Canada needs to be prepared in terms of data collection, sharing and linkage of case data with hospitalization and genomic data by learning from the difficulties encountered during the COVID-19 pandemic, along with the success in obtaining and linking case, hospitalization, vaccination and genomic data in countries such as the United Kingdom ((50)). In particular, Canada would benefit from building a framework of access to linked data for skilled experts, under appropriate conditions of access, well before it is next needed.

### The need for highly qualified personnel

A key lesson learned from the COVID-19 pandemic is that public health organizations need in-house HQP to be able to create models, bring together the multidisciplinary skills needed and conduct modelling of utility for public health purposes. Knowledge synthesis teams are crucial for incorporating rapidly evolving evidence into models; geographers and mathematicians are needed for importation, network and spread modelling; epidemiologists and medical, microbiology and immunology experts are required for ensuring biological reality; communication experts are necessary for explaining technical modelling results to the public; methods are needed for bringing these skills together ((10)). Explicit linkage of these HQP with modellers in academia provides opportunities for modelling within public health organizations to benefit from ongoing innovations, peer review, enhancement of modelling capacity, development of modelling ensemble approaches and transparency that enhances public confidence in the modelling being conducted ((60)). Without in-house modelling expertise, public health is unprepared to adequately respond to outbreaks and pandemics and must turn to external modellers to undertake the work. The availability and capability of external modellers would not be guaranteed, and without internal experts, public health would not be able to review or adapt the resulting models nor ensure that results are accurately communicated with decision-makers associated with loss of corporate memory of modelling.

### The need for a national community of practice of modellers

Many countries have recognized the importance of academic modellers contributing to public health decision-making during the COVID-19 pandemic ((47,61)). In the aftermath of the SARS-CoV-1 pandemic in 2003, a community of practice of infectious disease modellers formed in Canada and eventually became known as Pandemic Influenza Outbreak Research Modelling, or Pan-InfORM, in 2008 ((62)). This community aimed to support the use of modelling to inform decisions during pandemics. Although not specifically targeting pandemic preparedness per se, this community of practice did support decisions during the pH1N1 pandemic in Canada ((4,63)) and had links to public health organizations ((62)). While this group continued “peacetime” activities of modelling infectious disease transmission in collaboration with public health organizations up to 2018, between that time and the onset of the COVID-19 epidemic in Canada in 2020, links with most public health organizations had been lost, and new communities of practice, such as the Ontario Science Table and PHAC’s External Expert Modelling Group ((64)), had to be created in the face of the pandemic. The loss of Pan-InfORM as a recognized resource for public health in the face of COVID-19 underlines the need for public health organizations to have in-house HQP that can maintain collaborative modelling communities of practice outside of the times when we are responding to infectious disease emergencies.

## Modelling to supporting pandemic resilience

Modelling studies can support resilience of public health organizations to pandemics. Such modelling has general application for outbreak management and design of interventions using NPIs. A summary of ways that modelling can support development of resiliency is presented in **Table 1**.

**Table 1:** Examples of modelling studies that may support design of policies to increase resilience to pandemics

Focus area	Example modelling objectives	References
Building design	Enhancing greater ventilation to reduce respiratory pathogen transmission, particularly in locations where large numbers of people congregate.	((65,66))
Estimations to support decisions on public health capacity	Estimation of the surveillance effort needed to detect cases of emerging pathogens.	((67,68))
	Estimation of the test-and-trace effort needed to control transmission, in the absence of restrictive measures, according to different characteristics of pathogens and the diseases they cause.	((31,69))
Tools for strategic decisions	Criteria for determining if elimination of a pathogen in a particular jurisdiction would be successful, or if public health measures should aim simply to ”flatten the curve” to limit impacts on healthcare.	((70))
	Estimation of the likelihood of control by test-and-trace versus restrictive measures, according to characteristics of pathogens and the diseases they cause (*R*_0_ and proportions of cases with asymptomatic, presymptomatic or severe manifestations).	((71))
	Criteria for targeting NPIs to specific demographic or geographic sections of the population.	((32,72))
Best practices for use of public health measures	Best practices for the use of restrictive measures if these are needed to control transmission.	((52,73))
	Recommendations for the use of NPIs that reduce the probability or impact of transmission, such as distancing, masking and cohorting at gatherings.	((73,74,78))

Abbreviation: NPIs, non-pharmaceutical interventions

## Discussion

### Key challenges

Modelling requires computing infrastructure, software and mathematics, but it also requires the multidisciplinary teams of experts in all aspects of disease transmission and public health practice for the modelling to be grounded in the biological reality needed for decision-making in public health. Such teams were brought together in Canada during the COVID-19 pandemic, but they need to be maintained in some form to support future pandemic preparedness. An ongoing issue in Canada is the limitation of collection of granular data on disease cases, hospitalized cases, genomic characterization of causal agents and metadata that are crucial for analyses ((75,76)). Simultaneously, there is a current incapacity to link surveillance, hospital and genomic data across provinces and territories ((50)). These data issues are the subject of considerable efforts to remedy problems in collection, linkage and sharing within the Pan-Canadian Health Data Strategy ((77)) and, for health system data, in the Interoperability Roadmap of Canada Health Infoway ((78)), but they remain the most significant unresolved challenges to effective modelling of infectious diseases in Canada.

## Conclusion

Mathematical modelling of infectious diseases is now recognized as a key support to decision-making in public health preparedness and responses to outbreaks, epidemics and pandemics. Judicious use of modelling can support pandemic preparedness in terms of the stockpiles and planning needed to be prepared for a pandemic, while ready-to-go models, methods and HQP will support decision-making early in a pandemic. Modelling resources, particularly HQP, need to be maintained in public health organizations and in academia, and in transdisciplinary collaborative networks with public health-relevant scientists in other disciplines. A key barrier to effective modelling for public health decisions in Canada remains the issue of health data collection and sharing.

## References

[1] Wu JT, Cowling BJ. The use of mathematical models to inform influenza pandemic preparedness and response. Exp Biol Med (Maywood) 2011;236(8):955–61. 10.1258/ebm.2010.010271 21727183 PMC3178755

[2] Viboud C, Sun K, Gaffey R, Ajelli M, Fumanelli L, Merler S, Zhang Q, Chowell G, Simonsen L, Vespignani A; RAPIDD Ebola Forecasting Challenge group. The RAPIDD ebola forecasting challenge: synthesis and lessons learnt. Epidemics 2018;22:13–21. 10.1016/j.epidem.2017.08.002 28958414 PMC5927600

[3] World Health Organization. Non-pharmaceutical public health measures for mitigating the risk and impact of epidemic and pandemic influenza. Geneva, CH: WHO; 2019. https://www.who.int/publications/i/item/non-pharmaceutical-public-health-measuresfor-mitigating-the-risk-and-impact-of-epidemic-and-pandemic-influenza 42118904

[4] Fisman D; Pandemic Influenza Outbreak Research Modelling Team (Pan-InfORM). Modelling an influenza pandemic: A guide for the perplexed. CMAJ 2009;181(3-4):171–3. 10.1503/cmaj.090885 19620267 PMC2717691

[5] Tuite AR, Fisman DN, Kwong JC, Greer AL. Optimal pandemic influenza vaccine allocation strategies for the Canadian population. PLoS One 2010;5(5):e10520. 10.1371/journal.pone.0010520 20463898 PMC2865540

[6] Moghadas SM, Pizzi NJ, Wu J, Tamblyn SE, Fisman DN. Canada in the face of the 2009 H1N1 pandemic. Influenza Other Respir Viruses 2011;5(2):83–8. 10.1111/j.1750-2659.2010.00184.x 21306571 PMC4942003

[7] Greer AL, Schanzer D. Using a Dynamic Model to Consider Optimal Antiviral Stockpile Size in the Face of Pandemic Influenza Uncertainty. PLoS One 2013;8(6):e67253. 10.1371/journal.pone.0067253 23805303 PMC3689716

[8] World Health Organization. Prioritizing diseases for research and development in emergency contexts. Geneva, CH: WHO; 2024. https://www.who.int/activities/prioritizing-diseases-for-research-and-development-in-emergency-contexts

[9] Ogden NH, Bouchard C, Brankston G, Brown E, Corrin T, Dibernardo A. Infectious diseases. Health of Canadians in a Changing Climate: Advancing Our Knowledge for Action. Ottawa, ON: Health Canada; 2022. [Accessed 2024 Sept 10]. https://changingclimate.ca/health-in-a-changing-climate/

[10] Guillouzic S, MacLeod MR, Waller D, Bourdon S. How Flexible OR&A Teams Provided Decision Advantage through Pandemic Uncertainty. Proceedings of the 15th NATO Operational Research & Analysis Conference. 2021. https://cradpdf.drdc-rddc.gc.ca/PDFS/unc472/p815024_A1b.pdf#:~:text=How%20Flexible%20OR&A%20Teams%20Provided

[11] Becker AD, Grantz KH, Hegde ST, Bérubé S, Cummings DA, Wesolowski A. Development and dissemination of infectious disease dynamic transmission models during the COVID-19 pandemic: what can we learn from other pathogens and how can we move forward? Lancet Digit Health 2021;3(1):e41–50. 10.1016/S2589-7500(20)30268-5 33735068 PMC7836381

[12] Thompson J, McClure R, Scott N, Hellard M, Abeysuriya R, Vidanaarachchi R, Thwaites J, Lazarus JV, Lavis J, Michie S, Bullen C, Prokopenko M, Chang SL, Cliff OM, Zachreson C, Blakely A, Wilson T, Ouakrim DA, Sundararajan V. A framework for considering the utility of models when facing tough decisions in public health: a guideline for policy-makers. Health Res Policy Syst 2022;20(1):107. 10.1186/s12961-022-00902-6 36209122 PMC9547676

[13] Brooks RJ, Tobias AM. Choosing the best model: level of detail complexity and model performance. Math Comput Model 1996;24(4):1–14. 10.1016/0895-7177(96)00103-3

[14] Basu S, Andrews J. Complexity in mathematical models of public health policies: a guide for consumers of models. PLoS Med 2013;10(10):e1001540. 10.1371/journal.pmed.1001540 24204214 PMC3812083

[15] Li M, Dushoff J, Bolker BM. Fitting mechanistic epidemic models to data: A comparison of simple Markov chain Monte Carlo approaches. Stat Methods Med Res 2018;27(7):1956–67. 10.1177/0962280217747054 29846150 PMC6027774

[16] Vernon I, Owen J, Aylett-Bullock J, Cuesta-Lazaro C, Frawley J, Quera-Bofarull A, Sedgewick A, Shi D, Truong H, Turner M, Walker J, Caulfield T, Fong K, Krauss F. Bayesian emulation and history matching of JUNE. Philos Trans A Math Phys Eng Sci 2022;380(2233):20220039. 10.1098/rsta.2022.0039 35965471 PMC9376712

[17] Gallo L, Frasca M, Latora V, Russo G. Lack of practical identifiability may hamper reliable predictions in COVID-19 epidemic models. Sci Adv 2022;8(3):eabg5234. 10.1126/sciadv.abg5234 35044820 PMC8769547

[18] Bhattacharya P, Chen J, Hoops S, Machi D, Lewis B, Venkatramanan S, Wilson ML, Klahn B, Adiga A, Hurt B, Outten J, Adiga A, Warren A, Baek YY, Porebski P, Marathe A, Xie D, Swarup S, Vullikanti A, Mortveit H, Eubank S, Barrett CL, Marathe M. Data-driven scalable pipeline using national agent-based models for real-time pandemic response and decision support. Int J High Perform Comput Appl 2023;37(1):4–27. 10.1177/10943420221127034 38603425 PMC9596688

[19] Public Health Agency of Canada. COVID-19 in Canada: Using data and modelling to inform public health action. Ottawa, ON: PHAC; 2020. https://www.canada.ca/content/dam/phac-aspc/documents/services/diseases/2019-novel-coronavirus-infection/using-data-modelling-inform-eng.pdf

[20] Kleijnen JP. Verification and validation of simulation models. Eur J Oper Res 1995;82(1):145–62. 10.1016/0377-2217(94)00016-6

[21] Balci O. Verification, Validation, and Testing. In Handbook of Simulation. Wiley Online Library 1998. 10.1002/9780470172445.ch10 10.1002/9780470172445.ch10

[22] Pollett S, Johansson MA, Reich NG, Brett-Major D, Del Valle SY, Venkatramanan S, Lowe R, Porco T, Berry IM, Deshpande A, Kraemer MU, Blazes DL, Pan-Ngum W, Vespigiani A, Mate SE, Silal SP, Kandula S, Sippy R, Quandelacy TM, Morgan JJ, Ball J, Morton LC, Althouse BM, Pavlin J, van Panhuis W, Riley S, Biggerstaff M, Viboud C, Brady O, Rivers C. Recommended reporting items for epidemic forecasting and prediction research: the EPIFORGE 2020 guidelines. PLoS Med 2021;18(10):e1003793. 10.1371/journal.pmed.1003793 34665805 PMC8525759

[23] Milwid RM, Gabriele-Rivet V, Ogden NH, Turgeon P, Fazil A, London D, de Montigny S, Rees EE. A methodology for estimating SARS-CoV-2 importation risk by air travel into Canada between July and November 2021. BMC Public Health 2024;24(1):1088. 10.1186/s12889-024-18563-1 38641571 PMC11027292

[24] Tardy O, Vincenot CE, Bouchard C, Ogden NH, Leighton PA. Context-dependent host dispersal and habitat fragmentation determine heterogeneity in infected tick burdens: an agent-based modelling study. R Soc Open Sci 2022;9(3):220245. 10.1098/rsos.220245 35360357 PMC8965412

[25] Prem K, Cook AR, Jit M. Projecting social contact matrices in 152 countries using contact surveys and demographic data. PLOS Comput Biol 2017;13(9):e1005697. 10.1371/journal.pcbi.1005697 28898249 PMC5609774

[26] Mistry D, Litvinova M, Pastore Y Piontti A, Chinazzi M, Fumanelli L, Gomes MF, Haque SA, Liu QH, Mu K, Xiong X, Halloran ME, Longini IM Jr, Merler S, Ajelli M, Vespignani A. Inferring high-resolution human mixing patterns for disease modeling. Nat Commun 2021;12(1):323. 10.1038/s41467-020-20544-y 33436609 PMC7803761

[27] van den Hoogen J, Okazawa S. A Stochastic Model of COVID-19 Infections During a Large-Scale Canadian Army Exercise. The 15th NATO Operations Research & Analysis (OR&A) Conference Proceedings: Emerging and Disruptive Technology NATO STO Review. Ottawa, ON: National Defence; 2022. https://cradpdf.drdc-rddc.gc.ca/PDFS/unc399/p814956_A1b.pdf

[28] Okazawa S, van den Hoogen J, Guillouzic S. PyCoMod (Python Compartment Modelling) Programming Reference. Defence Research and Development Canada DRDC-RDDC-2023-D111. Ottawa, ON: Government of Canada; 2023. https://pubs.drdc-rddc.gc.ca/BASIS/pcandid/www/engpub/DDW?W%3DSYSNUM=817298&r=0

[29] Day T, Kennedy DA, Read AF, Gandon S. Pathogen evolution during vaccination campaigns. PLoS Biol 2022;20(9):e3001804. 10.1371/journal.pbio.3001804 36149891 PMC9553060

[30] Otto SP, Day T, Arino J, Colijn C, Dushoff J, Li M, Mechai S, Van Domselaar G, Wu J, Earn DJ, Ogden NH. The origins and potential future of SARS-CoV-2 variants of concern in the evolving COVID-19 pandemic. Curr Biol 2021;31(14):R918–29. 10.1016/j.cub.2021.06.049 34314723 PMC8220957

[31] Ng V, Fazil A, Waddell LA, Bancej C, Turgeon P, Otten A, Atchessi N, Ogden NH. Projected effects of nonpharmaceutical public health interventions to prevent resurgence of SARS-CoV-2 transmission in Canada. CMAJ 2020;192(37):E1053–64. 10.1503/cmaj.200990 32778573 PMC7513947

[32] Gabriele-Rivet V, Spence KL, Ogden NH, Fazil A, Turgeon P, Otten A, Waddell LA, Ng V. Modelling the impact of age-stratified public health measures on SARS-CoV-2 transmission in Canada. R Soc Open Sci 2021;8(11):210834. 10.1098/rsos.210834 34737875 PMC8562391

[33] Tardy O, Bouchard C, Chamberland E, Fortin A, Lamirande P, Ogden NH, Leighton PA. Mechanistic movement models reveal ecological drivers of tick-borne pathogen spread. J R Soc Interface 2021;18(181):20210134. 10.1098/rsif.2021.0134 34376091 PMC8355688

[34] Mohammadi Z, Cojocaru M, Arino J, Hurford A. Importation models for travel-related SARS-CoV-2 cases reported in Newfoundland and Labrador during the COVID-19 pandemic. medRxiv 2023; 23291136. 10.1101/2023.06.08.23291136 PMC12646790 41307065

[35] Hurford A, Martignoni MM, Loredo-Osti JC, Anokye F, Arino J, Husain BS, Gaas B, Watmough J. Pandemic modelling for regions implementing an elimination strategy. J Theor Biol 2023;561:111378. 10.1016/j.jtbi.2022.111378 36584747 PMC9794400

[36] du Plessis L, McCrone JT, Zarebski AE, Hill V, Ruis C, Gutierrez B, Raghwani J, Ashworth J, Colquhoun R, Connor TR, Faria NR, Jackson B, Loman NJ, O’Toole Á, Nicholls SM, Parag KV, Scher E, Vasylyeva TI, Volz EM, Watts A, Bogoch II, Khan K, Aanensen DM, Kraemer MU, Rambaut A, Pybus OG; COVID-19 Genomics UK (COG-UK) Consortium. Establishment and lineage dynamics of the SARS-CoV-2 epidemic in the UK. Science 2021;371(6530):708–12. 10.1126/science.abf2946 33419936 PMC7877493

[37] Murall CL, Fournier E, Galvez JH, N’Guessan A, Reiling SJ, Quirion PO, Naderi S, Roy AM, Chen SH, Stretenowich P, Bourgey M, Bujold D, Gregoire R, Lepage P, St-Cyr J, Willet P, Dion R, Charest H, Lathrop M, Roger M, Bourque G, Ragoussis J, Shapiro BJ, Moreira S. A small number of early introductions seeded widespread transmission of SARS-CoV-2 in Québec, Canada. Genome Med 2021;13(1):169. 10.1186/s13073-021-00986-9 34706766 PMC8550813

[38] McLaughlin A, Montoya V, Miller RL, Mordecai GJ, Worobey M, Poon AF, Joy JB; Canadian COVID-19 Genomics Network (CanCOGen) Consortium. Genomic epidemiology of the first two waves of SARS-CoV-2 in Canada. eLife 2022;11:e73896. 10.7554/eLife.73896 35916373 PMC9345601

[39] Nextstrain: Real-time tracking of pathogen evolution. [Accessed 2024 Aug 27]. https://nextstrain.org 10.1093/bioinformatics/bty407 PMC6247931 29790939

[40] Park SW, Sun K, Champredon D, Li M, Bolker BM, Earn DJ, Weitz JS, Grenfell BT, Dushoff J. Forward-looking serial intervals correctly link epidemic growth to reproduction numbers. Proc Natl Acad Sci USA 2021;118(2):e2011548118. 10.1073/pnas.2011548118 33361331 PMC7812760

[41] Dai X, Champredon D, Fazil A, Mangat CS, Peterson SW, Mejia EM, Lu X, Chekouo T. Statistical framework to support the epidemiological interpretation of SARS-CoV-2 concentration in municipal wastewater. Sci Rep 2022;12(1):13490. 10.1038/s41598-022-17543-y 35931713 PMC9355971

[42] Nourbakhsh S, Fazil A, Li M, Mangat CS, Peterson SW, Daigle J, Langner S, Shurgold J, D’Aoust P, Delatolla R, Mercier E, Pang X, Lee BE, Stuart R, Wijayasri S, Champredon D. A wastewater-based epidemic model for SARS-CoV-2 with application to three Canadian cities. Epidemics 2022;39:100560. 10.1016/j.epidem.2022.100560 35462206 PMC8993419

[43] Hellewell J, Abbott S, Gimma A, Bosse NI, Jarvis CI, Russell TW, Munday JD, Kucharski AJ, Edmunds WJ, Funk S, Eggo RM; Centre for the Mathematical Modelling of Infectious Diseases COVID-19 Working Group. Feasibility of controlling COVID-19 outbreaks by isolation of cases and contacts. Lancet Glob Health 2020;8(4):e488–96. 10.1016/S2214-109X(20)30074-7 32119825 PMC7097845

[44] Levesque J, Maybury DW, Shaw RH. A model of COVID-19 propagation based on a gamma subordinated negative binomial branching process. J Theor Biol 2021 Mar;512:110536. 10.1016/j.jtbi.2020.110536 33186594 PMC7654309

[45] Biron K, Drouin PL, Serre L. A branching process and simulation model to evaluate the spread of severe acute respiratory syndrome coronavirus 2 (SARS-Cov-2) in various environments: Enabling simultaneous mitigation strategies including social distancing masks symptomatic self isolation testing contact tracing and vaccination. Defence Research and Development Canada. Ottawa, ON: DRDC; 2023. https://pubs.drdc-rddc.gc.ca/BASIS/pcandid/www/engpub/DDW?W%3DSYSNUM=816419&r=0

[46] Volz E, Mishra S, Chand M, Barrett JC, Johnson R, Geidelberg L, Hinsley WR, Laydon DJ, Dabrera G, O’Toole Á, Amato R, Ragonnet-Cronin M, Harrison I, Jackson B, Ariani CV, Boyd O, Loman NJ, McCrone JT, Gonçalves S, Jorgensen D, Myers R, Hill V, Jackson DK, Gaythorpe K, Groves N, Sillitoe J, Kwiatkowski DP, Flaxman S, Ratmann O, Bhatt S, Hopkins S, Gandy A, Rambaut A, Ferguson NM; COVID-19 Genomics UK (COG-UK) consortium. Assessing transmissibility of SARS-CoV-2 lineage B.1.1.7 in England. Nature 2021;593(7858):266–9. 10.1038/s41586-021-03470-x 33767447

[47] Medley GF. A consensus of evidence: the role of SPI-M-O in the UK COVID-19 response. Adv Biol Regul 2022;86:100918. 10.1016/j.jbior.2022.100918 36210298 PMC9525209

[48] Yuan P, Tan Y, Yang L, Aruffo E, Ogden NH, Bélair J, Heffernan J, Arino J, Watmough J, Carabin H, Zhu H. Assessing transmission risks and control strategy for monkeypox as an emerging zoonosis in a metropolitan area. J Med Virol 2023;95(1):e28137. 10.1002/jmv.28137 36089815

[49] Corrin T, Ayache D, Baumeister A, Young K, Pussegoda K, Ahmad R, Waddell L. COVID-19 literature surveillance-A framework to manage the literature and support evidence-based decision-making on a rapidly evolving public health topic. Can Commun Dis Rep 2023;49(1):5–9. 10.14745/ccdr.v49i01a02 36815866 PMC9902036

[50] Colijn C, Earn DJ, Dushoff J, Ogden NH, Li M, Knox N, Van Domselaar G, Franklin K, Jolly G, Otto SP. The need for linked genomic surveillance of SARS-CoV-2. Can Commun Dis Rep 2022;48(4):131–9. 10.14745/ccdr.v48i04a03 35480703 PMC9017802

[51] Public Health Agency of Canada. Update on COVID-19 in Canada: Epidemiology and Modelling. Ottawa, ON: PHAC; 2021. https://www.canada.ca/content/dam/phac-aspc/documents/services/diseases-maladies/coronavirus-disease-covid-19/epidemiological-economic-research-data/update-covid-19-canada-epidemiology-modelling-20210903-en.pdf

[52] Ng V, Fazil A, Waddell LA, Turgeon P, Otten A, Ogden NH. Modelling the impact of shutdowns on resurging SARS-CoV-2 transmission in Canada. R Soc Open Sci 2021;8(5):210233. 10.1098/rsos.210233 34123390 PMC8190545

[53] McCabe R, Kont MD, Schmit N, Whittaker C, Løchen A, Walker PG, Ghani AC, Ferguson NM, White PJ, Donnelly CA, Watson OJ. Communicating uncertainty in epidemic models. Epidemics 2021;37:100520. 10.1016/j.epidem.2021.100520 34749076 PMC8562068

[54] Betti MI, Abouleish AH, Spofford V, Peddigrew C, Diener A, Heffernan JM. COVID-19 Vaccination and Healthcare Demand. Bull Math Biol 2023;85(5):32. 10.1007/s11538-023-01130-x 36930340 PMC10021065

[55] Public Health Agency of Canada. National Emergency Strategic Stockpile (NESS). Ottawa, ON: PHAC; 2024. [Accessed 2024 Sept 10]. https://www.canada.ca/en/public-health/services/emergency-preparedness-response/national-emergency-strategic-stockpile.html

[56] Meurens F, Dunoyer C, Fourichon C, Gerdts V, Haddad N, Kortekaas J, Lewandowska M, Monchatre-Leroy E, Summerfield A, Wichgers Schreur PJ, van der Poel WH, Zhu J. Animal board invited review: risks of zoonotic disease emergence at the interface of wildlife and livestock systems. Animal 2021;15(6):100241. 10.1016/j.animal.2021.100241 34091225 PMC8172357

[57] Mollentze N, Streicker DG. Predicting zoonotic potential of viruses: where are we? Curr Opin Virol 2023;61:101346. 10.1016/j.coviro.2023.101346 37515983

[58] World Health Organization. WHO R&D Blueprint for Epidemics Updating the WHO list of pathogens with epidemic and PHEIC potential. Geneva, CH: WHO; 2022. [Accessed 2024 Sept 10]. https://cdn.who.int/media/docs/default-source/blue-print/rd-blueprint_prioritization-2022_concept-note_v.1.pdf?sfvrsn=260e4e8f_3

[59] Milwid RM, Li M, Fazil A, Maheu-Giroux M, Doyle CM, Xia Y, Cox J, Grace D, Dvorakova M, Walker SC, Mishra S, Ogden NH. Exploring the dynamics of the 2022 mpox outbreak in Canada. J Med Virol 2023;95(12):e29256. 10.1002/jmv.29256 38054533

[60] Lewis MA, Brown P, Colijn C, Cowen L, Cotton C, Day T, Deardon R, Earn D, Haskell D, Heffernan J, Leighton P, Murty K, Otto S, Rafferty E, Hughes Tuohy C, Wu J, Zhu H. Charting a future for emerging infectious disease modelling in Canada. 2023. https://dspace.library.uvic.ca/server/api/core/bitstreams/5bd170ff-aa27-4fdf-af36-6e341c3749d8/content

[61] Jit M, Cook AR. Informing Public Health Policies with Models for Disease Burden, Impact Evaluation, and Economic Evaluation. Annu Rev Public Health 2024;45(1):133–50. 10.1146/annurev-publhealth-060222-025149 37871140

[62] Tariq M, Haworth-Brockman M, Moghadas SM. Ten years of Pan-InfORM: modelling research for public health in Canada. AIMS Public Health 2021;8(2):265–74. 10.3934/publichealth.2021020 34017890 PMC8116193

[63] Gojovic MZ, Sander B, Fisman D, Krahn MD, Bauch CT. Modelling mitigation strategies for pandemic (H1N1) 2009. CMAJ 2009;181(10):673–80. 10.1503/cmaj.091641 19825923 PMC2774362

[64] Bhatia D, Allin S, Di Ruggiero E. Mobilization of science advice by the Canadian federal government to support the COVID-19 pandemic response. Humanit Soc Sci Commun 2023;10(1):19. 10.1057/s41599-023-01501-8 36687774 PMC9844194

[65] Yan S, Wang L, Birnkrant MJ, Zhai Z, Miller SL. Multizone modeling of airborne SARS-CoV-2 quanta transmission and infection mitigation strategies in office hotel retail and school buildings. Buildings 2023;13(1):102. 10.3390/buildings13010102

[66] World Health Organization. Airborne Risk Indoor Assessment. Geneva, CH: WHO; 2024. [Accessed 2024 Sept 10]. https://partnersplatform.who.int/aria

[67] Champredon D, Fazil A, Ogden NH. Simple mathematical modelling approaches to assessing the transmission risk of SARS-CoV-2 at gatherings. Can Commun Dis Rep 2021;47(4):184–94. 10.14745/ccdr.v47i04a02 34035664 PMC8127697

[68] Rees EE, Rodin R, Ogden NH. Population surveillance approach to detect and respond to new clusters of COVID-19. Can Commun Dis Rep 2021;47(56):243–50. 10.14745/ccdr.v47i56a01 34220348 PMC8219061

[69] Ludwig A, Berthiaume P, Orpana H, Nadeau C, Diasparra M, Barnes J, Hennessy D, Otten A, Ogden N. Assessing the impact of varying levels of case detection and contact tracing on COVID-19 transmission in Canada during lifting of restrictive closures using a dynamic compartmental model. Can Commun Dis Rep 2020;46(1112):409–21. 10.14745/ccdr.v46i1112a08 33447163 PMC7799879

[70] Martignoni MM, Arino J, Hurford A. Is SARS-CoV-2 elimination or mitigation best? Regional and disease characteristics determine the recommended strategy. R Soc Open Sci 2024;11(6):240186. 10.1098/rsos.240186 39100176 PMC11295893

[71] Tupper P, Otto SP, Colijn C. Fundamental limitations of contact tracing for COVID-19. Facets 2021;6:1993–2001. 10.1139/facets-2021-0016

[72] Soucy JR, Ghasemi A, Sturrock SL, Berry I, Buchan SA, MacFadden DR, Brown KA. Trends in Interregional Travel to Shopping Malls and Restaurants Before and After Differential COVID-19 Restrictions in the Greater Toronto Area. JAMA Netw Open 2021;4(8):e2123139. 10.1001/jamanetworkopen.2021.23139 34463749 PMC8408668

[73] Hongoh V, Maybury D, Levesque J, Fazil A, Otten A, Turgeon P, Waddell L, Ogden NH. Decision analysis support for evaluating transmission risk of COVID-19 in places where people gather. Can Commun Dis Rep 2021;47(11):446–60. 10.14745/ccdr.v47i11a02 34880707 PMC8601102

[74] Hoffman B, Gaas B, McPhee-Knowles S, Guillouzic S, Kanary L. Development of an age-adjusted activity-based contact probability model for infectious diseases. Facets 2024;9:1–11. 10.1139/facets-2023-0094

[75] Wolfson M. COVID-19 data and modeling: we need to learn from and act on our experiences. Can J Public Health 2024;115(4):535–40. 10.17269/s41997-024-00917-2 39060712 PMC11382642

[76] Xia Y, Flores Anato JL, Colijn C, Janjua N, Irvine M, Williamson T, Varughese MB, Li M, Osgood N, Earn DJ, Sander B, Cipriano LE, Murty K, Xiu F, Godin A, Buckeridge D, Hurford A, Mishra S, Maheu-Giroux M. Canada’s provincial COVID-19 pandemic modelling efforts: A review of mathematical models and their impacts on the responses. Can J Public Health 2024;115(4):541–57. 10.17269/s41997-024-00910-9 39060710 PMC11382646

[77] Public Health Agency of Canada. Working with partners to modernize public health data. Ottawa, ON: PHAC; 2024. [Accessed 2024 Sept 10]. https://www.canada.ca/en/public-health/programs/pan-canadian-health-data-strategy.html

[78] Canada Health Infoway. Shared Pan-Canadian Interoperability Roadmap. 2023. [Accessed 2024 Sept 10]. https://www.infoway-inforoute.ca/en/component/edocman/6444-connecting-you-to-modern-health-care-shared-pan-canadian-interoperability-roadmap/view-document?Itemid=101

